# Decolourization of azo dyes by a newly isolated *Klebsiella* sp. strain Y3, and effects of various factors on biodegradation

**DOI:** 10.1080/13102818.2014.926053

**Published:** 2014-07-10

**Authors:** Daizong Cui, Guofang Li, Min Zhao, Song Han

**Affiliations:** ^a^ Department of Microbiology, College of Life Science, Northeast Forestry University, Harbin, P. R. China

**Keywords:** azo dyes, *Klebsiella* sp., decolourization, biodegradation

## Abstract

In this study, we isolated and characterized a new strain of *Klebsiella* sp. Y3, which was capable of decolourizing azo dyes under anaerobic conditions. The effects of physico-chemical parameters on the Methyl Red degradation by the strain were determined. The results indicated that strain Y3 exhibited a good decolourization ability in the range of pH from 4 to 9, temperature from 30 °C to 42 °C and salinity from 1% to 4%. A broad spectrum of azo dyes with different structures could be decolourized by the strain. The isolate decolourized Methyl Red, Congo Red, Orange I and Methyl Orange by almost 100% (100 mg/L) in 48 h. The culture exhibited an ability to decolourize repeated additions of dye, showing that the strain could be used for multiple cycles of biodegradation. Azo dyes at high concentrations could be tolerated and degraded by Y3. An almost complete mineralization of Methyl Red and Congo Red at the concentration of 800 mg/L was observed within 48 h. The high degradation potential of this bacterium supports its use in the treatment of industrial wastewater containing azo dyes.

## Introduction

Azo dyes, characterized by one or more azo bonds (–N = N–), are a major group of dyestuffs used in printing, textile, cosmetics, food and other industries.[1] However, during the dyeing process, up to 15% of the dyestuffs are discharged into the environment.[2] Most azo dyes and their metabolites may be carcinogens and mutagens, and they are not readily degradable in acidic and alkaline conditions and are resistant to temperature and light.[3,4] Thus, industrial wastewater containing azo dyes must be treated before it is released into the environment.[5]

Many new processes for azo dye decolourization have been developed. One promising strategy is the use of microbes to decolourize azo dyes.[6] The biodegradation of azo dyes is considered to be an environmentally friendly option.[7] In recent years, several phylogenetically diverse bacteria have been shown to be capable of reducing azo dyes under both anaerobic and aerobic conditions, including obligate anaerobes such as *Clostridium* sp. and *Eubacterium* sp.[8]; facultative anaerobes such as *Enterobacter agglomerans*, *Escherichia coli* and *Bacillus cereus* [9–11]; and some aerobes such as *Pseudomonas aeruginosa*.[12] There could be summarized two different mechanisms for bacterial azo dye decolourization.[13] Aerobic bacteria usually show a high specificity to dye structures and need to be acclimatized over a long period in the presence of azo compounds to induce azoreductase expression. In contrast, anaerobic decolourization is usually unspecific, and the anaerobic dye removal efficiency is much higher than that of aerobic decolourization. These findings indicate that the anaerobic process is more useful for decolourizing a broad range of azo dyes in wastewater.[4]

In this study, we isolated a bacterium, *Klebsiella* sp. strain Y3, that could degrade a wide range of azo dyes under anaerobic conditions. To obtain more details about the anaerobic decolourization process, various parameters such as temperature, pH, salinity and different initial dye concentrations were investigated. The results showed that strain Y3 had a high ability to decolourize azo dyes and had a great potential for use in the treatment of effluents containing azo dyes.

## Materials and methods

### Dyes and chemicals

**Figure 1.**
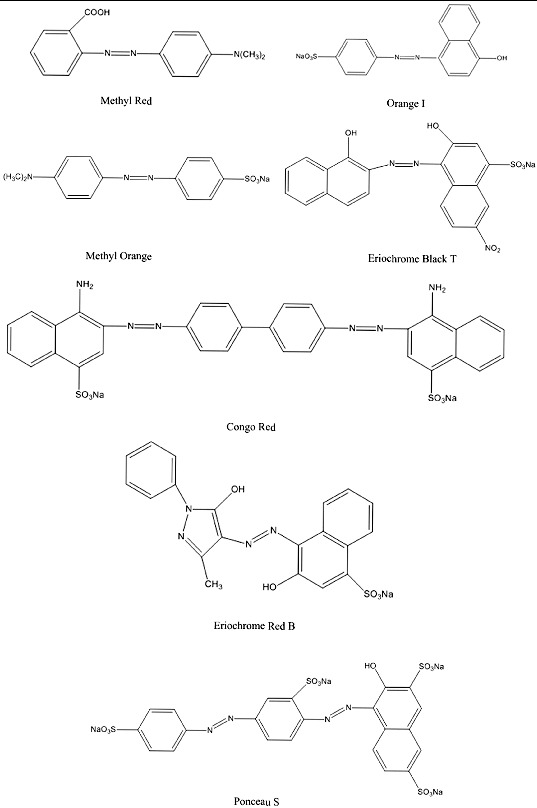
Chemical structures of azo dyes used in this study.

The dyes used in the study were of industrial grade and were procured from the Guangfu Fine Chemical Research Institute (Tianjin, China). The dyes tested were Methyl Red, Congo Red, Orange I, Methyl Orange, Eriochrome Red B, Eriochrome Black T and Ponceau S. The chemical structures of the dyes are shown in Figure 1. The rTaq DNA polymerase, dNTPS, PCR purification kit, pMD-18T vector and *E.coli* JM109 strain were purchased from TaKaRa (Dalian, China). All of the other chemicals used were of analytical grade.

### Strain isolation and identification

The wastewater used to isolate dye-decolourizing bacterial cultures was collected from a textile-dyeing industry in Haicheng, China. For the enrichment of the dye-decolourizing strains, 75 mL serum bottles containing Luria–Bertani (LB) culture media supplemented with 50 mg/L of Methyl Red were inoculated with 1 mL of contaminated water. After flushing with nitrogen gas for 5 min, the serum bottles were incubated at 37 °C for 16 h in an anaerobic incubation chamber (YQX-Ⅱ, CIMO medical instrument manufacturing Co., LTD, China). Then, 1 mL of each culture was transferred to 75 mL of LB culture medium containing 100 mg/L Methyl Red and incubated at 37 °C for another 16 h. Stable enrichment cultures were obtained after the subculture step. The mineral salts medium (MSM) used for the isolation and decolourization studies contained the following ingredients: 10 g/L mannitol, 5 g/L NH_4_Cl, 5 g/L KH_2_PO_4_, 5 g/L Na_2_HPO_4_ and 100 mg/L Methyl Red. The screening of microorganisms for azo dye decolourization was carried out on agar plates. The different strains were isolated and the one with the highest dye degradation ability was used for further study.

The isolated strain was identified by using 16S rDNA gene sequence analysis. The genomic DNA extraction of the isolated culture was carried out as described previously.[14] The 16S rDNA gene was amplified by polymerase chain reaction (PCR) using the specific primers 27f (5′-GAGTTTGATCMTGGCTCAG-3′) and 1492r (5′-GGTTACCTTGTTACGACTT-3′). The PCR amplification used the following protocol: initial denaturation of DNA for 3 min at 94 °C; 30 cycles of 1 min at 94 °C, 1 min at 56 °C and 1.5 min at 72 °C, followed by a final extension for 10 min at 72 °C. The amplified product was purified and was cloned into the pMD18-T vector. The DNA sequences were determined using the chain-termination method on an ABI 3730 DNA sequencer by a commercial service (Sangon China). The sequence was analysed on the National Center for Biotechnology Information (NCBI) server, using the BLAST tool, and the corresponding sequences were downloaded. The DNA sequences were aligned using the Clustal X 1.81 program. Phylogenetic analyses were performed by using programs in the Phylip 3.67 software package. The sequence has been deposited in GenBank with the accession number JN049592.

### Effects of various factors on Methyl Red decolourization

MSM media containing 100 mg/L of Methyl Red and one of a number of additions as indicated below were incubated with the strain under anaerobic conditions.

Effects of temperature: The cultures were incubated at various temperatures (5–55 °C).

Effects of pH: The reaction mixtures were adjusted to pH values between 3 and 11 by using 1 mol/L HCl and NaOH.

Effects of salinity: Various concentrations (1%–5%) of NaCl were added to the media.

### Decolourization of different azo dyes

Strain Y3 was checked for its ability to decolourize other azo dyes with different Chemical structures (Methyl Red, Congo Red, Orange I, Methyl Orange, Eriochrome Red B, Eriochrome Black T and Ponceau S). The initial concentration of each dye was adjusted to 100 mg/L. The degradation system was incubated with strain Y3 at 37 °C under anaerobic conditions.

### Repeated-batch decolourization test

Repeated batch decolourization test was conducted to indicate whether long-term degradation could be stably attained. First, the strain was inoculated into the MSM medium with 50 mg/L Methyl Red for static incubation at 37 °C. After 16 h, fresh Methyl Red was added to the culture to achieve a dye concentration of 50 mg/L for the next cycle. Several cycles were repeated to have a complete figure of long-term decolourization.

### Effect of initial dye concentration

The effect of different initial concentrations of Methyl Red and Congo Red ranging from 200 mg/L to 800 mg/L in MSM broth on the decolourization potential of the strain Y3 was also tested.

### UV–vis analysis

The dye degradation products produced during biodegradation after incubation under anaerobic conditions were studied by following the change in the ultraviolet–visible (UV–vis) spectra (from 200 nm to 800 nm), using a UV–vis spectrophotometer (Unico UV-2800, USA).

### Analytical methods

The samples were taken at different time intervals and analysed for decolourization and growth. The dye decolourization was estimated by measuring the absorbance at the respective λ_max_ of the different dyes individually in a UV–vis spectrophotometer. The rate of decolourization was calculated from the difference between initial and final absorbance values. The growth was monitored spectrophotometrically, and the absorbance of the culture broth was measured at 600 nm. All of the experiments were performed in triplicate.

## Results and discussion

### Identification of the isolated strain

**Figure 2.**
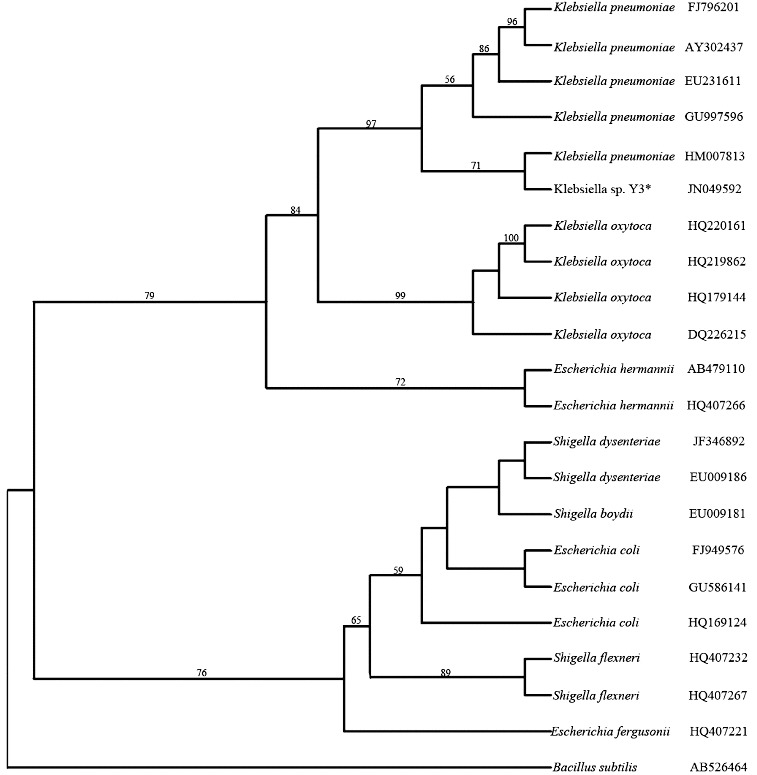
Consensus tree obtained by the maximum parsimony method based on the 16S rDNA sequences. *Bacillus subtilis* was used as an outgroup to root the tree. Bootstrap values, more than 50% from 1000 replications, were shown on the branches.

The isolates were selected based on their ability to form a high dye decolourization zone on an agar plate under anaerobic conditions. Among the seven isolated bacterial strains, one strain was selected based on its ability to decolourize azo dyes. This selected strain was identified on the basis of its 16S rDNA sequence. A 1503 bp fragment was amplified from the strain. After the initial analysis using the NCBI database, the relevant sequences were downloaded. A phylogenetic tree of the partial sequences based on the 16S rDNA was constructed by using the maximum parsimony method in the program Phylip 3.67 (Figure 2). *Bacillus subtilis* was used as an outgroup. In the tree, the isolate clustered with *Klebsiella pneumoniae*, and the bootstrap value was 97%. The results indicated that the isolate is a close relative to the genus *Klebsiella* and the strain was designated as *Klebsiella* sp. strain Y3.

*Klebsiella* is genus of Gram-negative, facultatively anaerobic bacteria of the family Enterobacteriaceae. In previous studies, many species in this family were reported to have azo dye-decolourizing abilities, including the species *Shigella dysenteriae*, *Proteus mirabilis*, *Enterobacter agglomerans* and *Escherichia coli*.[9,11,15,16] However, there are only a few studies reporting azo dye decolourization by *Klebsiella* sp. For example, Wong and Yuen [17] reported that the strain *Klebsiella pneumoniae* RS-13, which was isolated from dye-contaminated sludge, could completely decolourize Methyl Red (100 mg/L) under aerobic conditions. Franciscon et al. [18] showed that the *Klebsiella* sp. strain VN-31 could decolourize four different azo dyes under microaerophilic conditions. In our previous study [19], two decolourization consortia under aerobic and anaerobic conditions were developed, and denaturing gradient gel electrophoresis (DGGE) analysis of the two consortia revealed that *Klebsiella* was the prevalent genus in both aerobic and anaerobic conditions. The results showed that *Klebsiella* Sp. might have a great potential for azo dye degradation.

### Effect of temperature on dye decolourization

**Figure 3.**
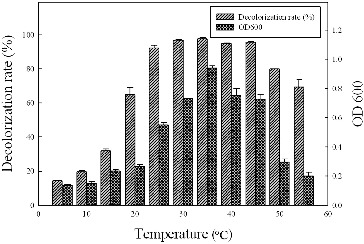
Effect of temperature on dye decolourization by strain Y3. The strain was anaerobically cultured in MSM synthetic medium containing 100 mg/L Methyl Red at pH 7.0 for 16 h. All assays were done in triplicate.

In this study, the temperature range of 5–55 °C was arranged for the strain growth and dye decolourizing tests (Figure 3). The optimum temperature for the dye decolourization was 37 °C, at which over 97% of Methyl Red was degraded within 16 h. The decolourized fraction remained above 94% at 25–45°C; however, it dropped dramatically after the temperature was decreased to below 20 °C. Only 15% of Methyl Red was decolourized when the temperature dropped to 5 °C. The growth rate decreased greatly when the temperature was 50 °C, although more than 65% of Methyl Red was still decolourized at that temperature.

Strain Y3 exhibited a good decolourization ability in a wide temperature range. The results showed some deviation from the findings of other studies. The *Klebsiella pneumoniae* strain RS-13, which was isolated by Wong et al.[17] could not degrade Methyl Red at 45 °C because of its poor ability to grow at high temperatures. Chang et al. [20] reported that the decolourization rate of the strain *Pseudomonas luteola* dropped dramatically after the temperature increased to over 40 °C.

### Effect of pH on dye decolourization

**Figure 4.**
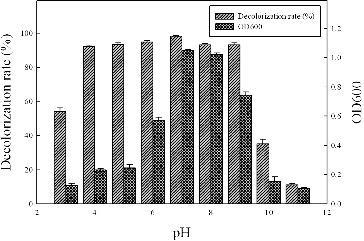
Effect of pH on the dye decolourization of strain Y3. The strain was anaerobically cultured in MSM synthetic medium containing 100 mg/L Methyl Red at 37 °C for 16 h. All assays were done in triplicate.

The effects of pH on dye decolourizing were determined over a wide pH range from 3 to 11 (Figure 4). The results showed that changes in pH from 4 to 9 did not affect the fraction of decolourized dye, which remained at approximately 95%. The optimum pH for dye decolourization was 7.0, at which over 98% of the dye was decolourized within 16 h. Both the cell mass and the decolourization rate decreased at high pH values, and when the pH value reached 11, only approximately 10% of Methyl Red was degraded after the 16 h incubation. However, azo dyes could be decolourized by strain Y3 in acidic conditions, and more than 50% of Methyl Red was decolourized even at pH 3.0.

The majority of the azo dye-decolourizing species reported are able to degrade dyes at pH values near neutrality. *Klebsiella pneumoniae* RS-13 was found to completely degrade Methyl Red at pH of 6.0 to 8.0.[17] Modi et al. [10] found that pH between 6 and 7.5 was optimal for the decolourization of Reactive Red 195 by *Bacillus cereus* strain M1. A decrease in the initial pH has been shown to have a negative impact on the decolourization of azo dyes.[21] The negative impact likely occurred because azo bonds might include protons and form protonated azo dyes at low pH; thus, bacteria cannot decolourize azo dyes due to this alteration in chemical structures.[22] However, in our study, over 50% decolourization of Methyl Red was achieved at pH as low as 3, which is, to our knowledge, the lowest pH value at which the bacteria could grow and decolourize azo dyes.

### Effect of salinity on decolourization

**Figure 5.**
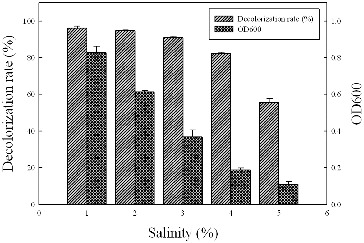
Effect of salinity on the dye decolourization of strain Y3. The strain was anaerobically cultured in MSM synthetic medium containing 100 mg/L Methyl Red at 37 °C at pH 7.0 for 16 h. All assays were done in triplicate.

Strain Y3 was also tested for its ability to decolourize Methyl Red in the presence of NaCl (Figure 5). It was shown that the growth rate of Y3 decreased at high salt concentrations; however, the decolourization rate maintained above 80% at 1%–4% salt concentrations. Similar results have been reported by Kolekar et al.[23] who showed that *Bacillus fusiformis* strain KMK5 could decolourize Acid Orange 10 effectively within 48 h in 0.5–3% salt concentrations. The ability of Y3 to degrade Methyl Red even at high salt concentrations indicated that wastewater containing high amounts of salt could be treated by Y3.

### Decolourization of different azo dyes by strain Y3

**Figure 6.**
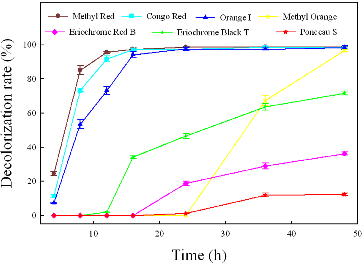
The ability of strain Y3 to decolourize different azo dyes. The strain was anaerobically cultured in MSM synthetic medium containing 100 mg/L of each dye at 37 °C at pH 7.0. All assays were done in triplicate.

Strain Y3 was tested for its ability to decolourize different azo dyes under anaerobic conditions. The results showed that the maximum percentage of decolourization and the degradation time varied among the different dyes (Figure 6). Almost 100% of Methyl Red, Congo Red, Orange I was decolourized in 24 h, whereas 73% of Eriochrome Black T was decolourized in 48 h. The decolourization of Methyl Orange was not obvious after the first 24 h, but the decolourization rate increased dramatically between 24 and 48 h, and more than 96% of Methyl Orange was degraded after 48 h. For Eriochrome Red B and Ponceau S, only 37% and 13% decolourization, respectively, were attained within 48 h. The results showed that both of these dyes were resistant to biodegradation in this study.

It has been reported that the different efficiencies of various azo dyes decolourization are greatly due to the chemical structures of the azo dyes.[24] The dyes with simple structures and low molecular weights are decolourized faster than those with complex structures.[13] In the present study, the three dyes (Methyl Red, Orange I and Methyl Orange) with simple structures had higher decolourization rates and shorter decolourization times than the dyes with complex structures (Eriochrome Red B and Ponceau S). The presence of sulfonates in azo dyes might introduce resistance to dye decolourization.[8] In our study, strain Y3 was able to decolourize dyes that contain one or two sulfonate groups (Methyl Orange, Congo Red, Orange I and Eriochrome Black T). However, Ponceau S has four sulfonate groups, and these structures may decrease the percentage of decolourization. The low decolourization rate for Eriochrome Red B was probably due to the pyrazole group beside the azo bond, which is more resistant to degradation than the benzene and naphthalene structures.

### Decolourization with repeated addition of dye aliquots

**Figure 7.**
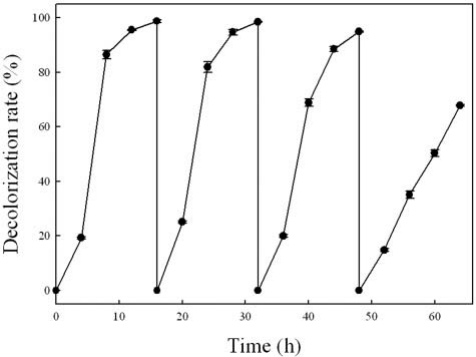
Repeated decolourization of Methyl Red (50 mg/L) under anaerobic condition.

Further studies were carried out to test the ability of strain Y3 to decolourize repeated additions of Methyl Red dye aliquots under anaerobic conditions (Figure 7). After 98% decolourization of the first dye aliquot within 16 h, a second dye aliquot was added, which was also 98% decolourized within the next 16 h. In another 16 h of incubation, the culture caused 95% decolourization of a third addition. However, a fourth dye aliquot addition caused a decrease in the decolourization rate, and only 68% of Methyl Red was degraded after 16 h of incubation. The eventual decrease in decolourization was likely due to nutrient depletion. After an addition of fresh medium, the colour removal was recovered during a fifth cycle. Over 90% of dye decolourization was observed in the next four cycles. These observations showed that strain Y3 had the ability to decolourize repeated additions of dye aliquots.

### Effect of initial dye concentration

The decolourization rate of Methyl Red and Congo Red by strain Y3 at different initial concentrations (200–800 mg/L) was studied (Table 1). The results showed that strain Y3 could decolourize both of these azo dyes effectively at varying initial dye concentrations. Over 95% decolourization of Methyl Red was observed within 16 h, 18 h, 24 h and 30 h for the dye concentrations of 200 mg/L, 400 mg/L, 600 mg/L and 800 mg/L, respectively. At 200 mg/L, 400 mg/L and 600 mg/L of Congo Red, over 90% of the dye was degraded within 24 h, 30 h and 36 h, respectively. However, when the Congo Red concentration was increased up to 800 mg/L, a slight reduction in the decolourization rate was observed, and only 85% of the dye was decolourized after a 48 h incubation. At the same time, a decrease in the culture growth at high concentrations of Congo Red was also observed in this study. The growth of the culture was found to be decreased by 30% as the dye concentration increased from 200 to 800 mg/L. The results indicated that high concentrations of dye were toxic and inhibited the growth of the bacterium.

**Table 1.** Decolourization of azo dyes at different concentrations by *Klebsiella* sp. strain Y3.

Dyes	Dye concentration (mg/L)	Incubation time (h)	Decolourization rate (%)
Methyl Red	200	16	98.56 ± 0.44
	400	18	97.63 ± 1.36
	600	24	97.61 ± 0.86
	800	30	97.01 ± 1.02
Congo Red	200	24	93.04 ± 1.16
	400	30	91.93 ± 0.67
	600	36	90.43 ± 0.31
	800	48	85.68 ± 2.80

In recent years, many bacteria have been reported to be capable of reducing various azo dyes under both anaerobic and aerobic conditions.[25] Among these dye-decolourizing bacteria, strain Y3 had a relatively high ability to tolerate azo dyes at high concentrations, and the decolourization efficiency of Y3 was much higher than that of other bacterial strains. The *Klebsiella* sp. strain VN-31 could completely decolourize 100 mg/L of Reactive Yellow 107 in periods of more than 68 h.[18] Parshetti et al. [26] reported that the percentage of decolourization of Methyl Orange by a *Kocuria rosea* strain decreased with increasing the dye concentration; only 40% of the dye (100 mg/L) was decolourized after 120 h of incubation. Tony et al. [27] found that the Congo Red decolourization rate decreased from 62.6% to 27.6% as the concentration of the dye was increased from 30 to 100 mg/L. However, strain Y3, which was isolated in this study, could degrade most of the added Methyl Red and Congo Red (800 mg/L) within 30 h and 48 h, respectively. The great potential of strain Y3 to decolourize azo dyes at high concentrations is significant for its commercial application.

### UV–vis characterization

**Figure 8.**
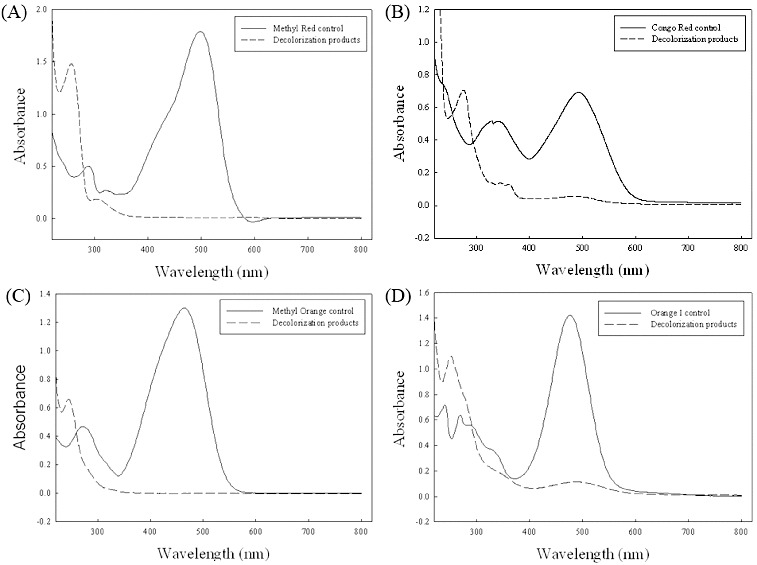
UV–vis spectra of the azo dyes before (solid lines) and after (dashed line) biodegradation: Methyl Red (**A**); Congo Red (**B**); Methyl Orange (**C**); Orange I (**D**).

In UV–vis analysis the azo dyes (Figure 8) showed that the λ_max_ for each dye was 498 nm, 500 nm, 465 nm and 476 nm for Methyl Red, Congo Red, Methyl Orange and Orange I, respectively. However, as time went by, the maximum wavelength for the different azo dyes shifted from the visible light range towards the UV range. The complete disappearance of the absorbance peaks in the visible region for each dye indicated their complete decolourization. After decolourization, the appearance of a new peak in the UV spectra indicated the formation of other metabolites.

## Conclusions

In the present study, we isolated and identified a new dye-degrading strain, *Klebsiella* sp. strain Y3, from a site contaminated by a textile-dyeing industry. The strain exhibited good azo-dye-decolourization ability under anaerobic conditions. This bacterium completely degraded 100 mg/L of Methyl red within 16 h. The culture exhibited good decolourization ability at temperatures from 25 to 45 °C and pH values from 4 to 9. The isolate Y3 could also decolourize Methyl Red effectively in high salt concentrations (1%–4%). The ability of the strain to tolerate and decolourize azo dyes in a wide range of temperature, pH and salinity conditions gave it an advantage for potential use in the treatment of textile industry wastewater. Five out of seven tested dyes were effectively degraded within 48 h. The culture had the ability to decolourize Methyl Red in repeated additions, which is significant for its commercial application. Strain Y3 could also tolerate high concentrations of azo dyes: over 85% of Methyl Red and Congo Red at 800 mg/L were decolourized within 30 h and 48 h, respectively. Overall, the results suggested that strain Y3 has the ability to decolourize azo dyes under anaerobic conditions, and it has a great potential for wastewater treatment. 
